# Structural, electrical, and multiferroic characteristics of lead-free multiferroic: Bi(Co_0.5_Ti_0.5_)O_3_–BiFeO_3_ solid solution

**DOI:** 10.1039/c8ra02306a

**Published:** 2018-11-01

**Authors:** Nitin Kumar, Alok Shukla, Nripesh Kumar, R. N. P. Choudhary, Ajeet Kumar

**Affiliations:** Department of Physics, National Institute of Technology Mizoram Aizawl-796012 India nitinphysicskushawaha@gmail.com; Department of Physics, Siksha O Anusandhan (Deemed to be University) Bhubaneswar-751030 India; School of Physics, University of Hyderabad Hyderabad-500046 India

## Abstract

A solid solution of bismuth cobalt titanate [Bi(Co_0.5_Ti_0.5_)O_3_] and bismuth ferrite (BiFeO_3_) with a composition Bi(Co_0.40_Ti_0.40_Fe_0.20_)O_3_ (abbreviated as BCTF80/20) was synthesized *via* a cost effective solid-state technique. Phase identification and basic structural symmetry of the samples were determined by analyzing powder X-ray diffraction data. Field emission scanning electron micrograph (FE-SEM) and energy dispersive X-ray (EDX) spectra were analyzed to evaluate the micro-structural aspects (shape and size, distribution of grains) as well as a quantitative evaluation of the sample. The average crystallite (particle) and grain size were found to be ∼30 nm and ∼1–2 micron, respectively. The electrical parameters (dielectric constant, tangent loss, impedance, modulus, and conductivity) of as-synthesized material were obtained in a temperature range of 300 to 773 K and frequency range of 1 kHz and 1000 kHz. The strong correlation of microstructure (*i.e.*, grains, grain boundary, *etc.*) and electrical parameters of this material were observed. The frequency dependence of electrical impedance and modulus exhibited a deviation from an ideal Debye-like relaxation process. The dependence of dielectric relaxation mechanism on frequency and temperature is discussed in detail. The field dependent polarization (*P*–*E* hysteresis loop) of BCTF80/20 exhibited an enhanced value of remnant polarization as compared to that of BiFeO_3_ (referred as BFO). At room temperature (300 K), the magnetic hysteresis loop measurements also showed a significant improvement in the magnetization of BCTF80/20. Thus, based on these enhanced values of remnant polarization and magnetic parameters, we can assume that BCTF80/20 may be considered as a promising candidate for some new generations of electronic devices.

## Abstract 2

A solid solution of bismuth cobalt titanate [Bi(Co_0.5_Ti_0.5_)O_3_] and bismuth ferrite (BiFeO_3_) with a composition Bi(Co_0.40_Ti_0.40_Fe_0.20_)O_3_ (abbreviated as BCTF80/20) was synthesized *via* a cost effective solid-state technique.

## Graphical abstract

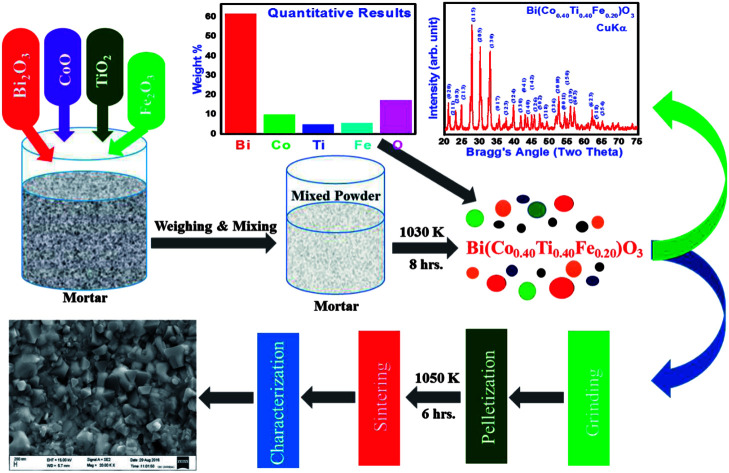

## 1. Introduction

Current research activities on lead-free based multiferroic systems have attracted much attention over the last few decades for the development of next generation electronic materials for devices such as transducers, magneto-electric sensors, memories with high storage capacity, and so on.^1–3^ Current convention employing the term ‘multiferroic’ has been formally defined as materials that combine more than one ferroic order parameters, namely ferroelectric, ferromagnetic/ferroelastic, simultaneously in a similar phase.^4^ Among all the multiferroic materials studied so far, BiFeO_3_ (abbreviated as BFO) is a perfect lead-free single-phase material that simultaneously possesses a lot of strong anti-ferromagnetic as well as ferroelectric features together with a high Neel temperature (*T*_N_ ∼ 643 K) and ferroelectric Curie temperature (*T*_FE_ ∼ 1103 K).^5^ The bulk BFO (perovskite-type) possesses a rhombohedral phase and its spontaneous polarization value is about ∼100 μC cm^−2^ in the (111) pseudo-cubic direction.^6–8^ At room temperature (300 K), it has unit cell lattice parameters, *a* = 3.965 Å, *α* = 89.46° with a space group of *R*3*c*.^9^ It seems that exceptions to the empirical assertion of the incompatibility of magnetism and ferroelectricity occurs, but really it is not an exception to the general statute, as the ferroelectricity mechanism is different from the formal one.^10^ In BFO, polarization primarily originates from the Bi^3+^ lone pair (A-site) and the magnetization mainly comes from Fe^3+^ (B-site). Its ferroelectric and magnetic features can be strongly influenced by A, B, or A/B-site doping. In spite of prominent advantages presented in this bismuth ferrite compound, its application for multiferroic devices still has been limited due to low resistivity and inhomogeneous spin symmetry (weak magnetic features).^11^ Many methods/sets of works on suitably doped or modified multiferroic materials (the enhancement of ferroelectric and magnetic properties) have been reported over the years. The doped/modified materials have enormous merits in several terms, like as an effective way to modulate epitaxial growth of superior thin films along with room temperature multiferroic characteristics. In this context, we tried to deliver a general doping perspective and assess its impact on material functionality. Single or multiple doping of tetravalent nonmagnetic ions, such as different compositions of Co/Ti into BFO, shows further enhancement of electrical and multiferroic properties.^12^

Research on the bismuth ferrite system shows some inherent problems, including high leakage current density and structural instability, which strongly affect the capacitive, resistive, and ferroelectric properties of the material. To solve the leakage current density and other related problems, several new research approaches have been tried, such as synthesis of a pure-phase system by substituting suitable ions/elements at different sites and fabricating solid solutions or composites. Nowadays, much attention is being paid to lead-free bismuth-based multiferroic solid solutions.^13^ Some recent developments show improvements in the multiferroic properties of co-substituted/modified BFO.^10,14–23^ It was expected that a solid solution of Bi(Co_0.5_Ti_0.5_)O_3_ and BiFeO_3_ in different ratios could significantly modify the multiferroic properties. In view of the importance of materials in the multiferroic family, we carried out a systematic study of the effects of frequency and temperature on dielectric, electrical, and magnetic characteristics of a lead-free solid solution with the composition Bi(Co_0.40_Ti_0.40_Fe_0.20_)O_3_; (hereafter BCTF80/20) which are reported in this paper.

## 2. Experimental techniques

### 2.1 Sample preparation

In this research, we used a solid-state reaction technique to synthesize a solid solution of bismuth ferrite and bismuth cobalt titanate with the chemical formula, Bi(Co_0.40_Ti_0.40_Fe_0.20_)O_3_ (referred as BCTF80/20). For this, a required amount of high-purity (>99.9% pure) oxide powders of bismuth tri-oxide, cobalt mono-oxide, titanium dioxide, and iron oxide were weighed using a high-precession digital balance followed by mixing them thoroughly. The mixture was ground in a mortar and pestle (fine powder) in dry air as well as in a wet medium (methanol) for eight hours. Then, the ground mixture was calcined with continuous heating at 1030 K for 8 hours. Prepared pellets of small dimension circular shape (diameter = 10 mm thickness = 1–2 mm) were sintered at a high temperature (1050 K) for 6 hours to produce a compact dense sample for electrical measurements.

### 2.2 Measurements and characterization

**Fig. 1:**
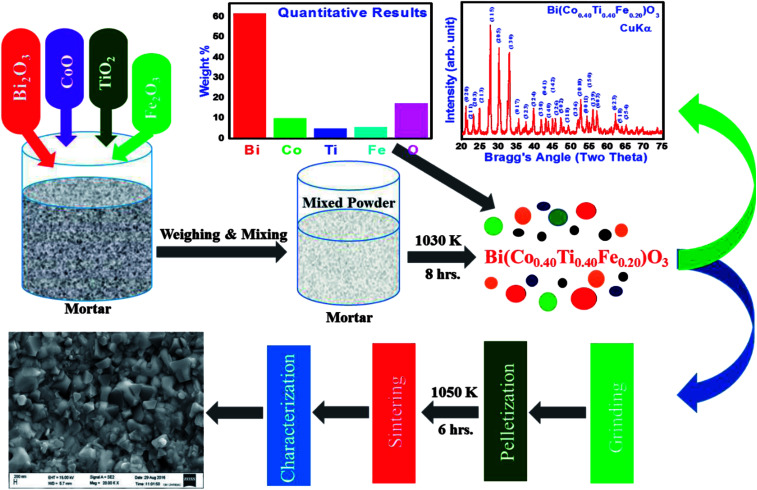
Schematic syntheses procedure and further characterization of Bi(Co_0.40_Ti_0.40_Fe_0.20_)O_3_ compound by solid state techniques.

Before pressing, the calcined powder the phase formation as well as the structure of specimen was required to be identified using X-ray diffraction data collected with the help of a Rigaku MiniFlex 600X-ray diffractometer in a wide range of Bragg angles (20° ≤ 2*θ* ≤ 75°). The “POUDMULT” software was employed to investigate crystal symmetry and lattice parameters. Room temperature Raman spectra of as prepared BCTF 80/20 were recorded using a micro-Raman system (LABRAM HR, Japan). Analysis of elements present in the required amount and microstructure of sintered pellets was carried out using energy-dispersive X-ray micro-analysis (EDXMA) and field emission scanning electron microscope (FESEM) techniques at room temperature (300 K). The ceramics sample was gold-coated before the FESEM measurement to prevent charge accumulation at the surface. For evaluating capacitive and resistance parameters, a high precision phase sensitive multimeter (PSM-1735) was used. We carried out said experiments in a wide range of frequencies (1 kHz to 1 MHz) and temperatures (300–773 K). The electrical parameters (impedance, modulus, and conductivity) were obtained using dielectric data. In order to examine the dielectric and electrical features of BCTF80/20, the calcined powder was pressed into circular shaped pellets of 12 mm diameter and 1.5 mm thickness under an iso-static pressure of 4.5 × 10^5^ kg cm^−2^ tons (KBr manual press), and then the pellets were sintered at an optimum temperature at 1050 K for six hours. The detailed measurement techniques used here were partially the same as those reported earlier for the development of lead-free other multifunctional materials.^9–12^ A detailed description of the sample preparation by a solid-state reaction technique and its characterization is summarized in Fig. 1.

The dielectric and electrical measurements of sintered pellets were carried out with a silver electrode (parallel surfaces) and annealed at 430 K for two hours to eliminate any moisture from them. These measurements were performed and recorded at same heating rate of 1 °C at a continuous rate of 5 to 25 K, respectively. Using both a vibrating sample magnetometer (M/S Lake Shore VSM-7410) and a hysteresis loop Analyzer (Radiant Precision Premier II *P*–*E* loop), the multiferroic properties (magnetization and polarization) of the samples were recorded at 300 K (room temperature).

## 3. Results and discussion

### 3.1 Structure and molecular structural studies

**Fig. 2:**
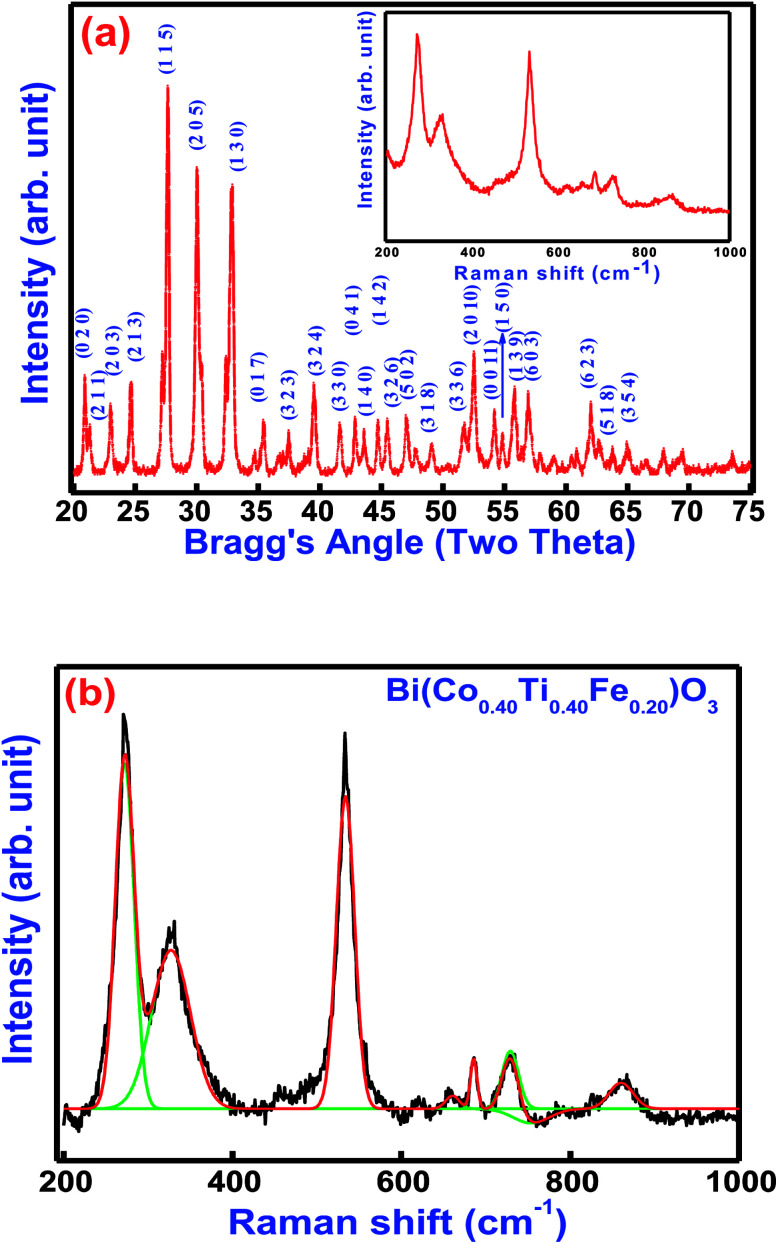
(a) Room temperature XRD pattern and inset shows Raman spectra and (b) Gaussian fitted Raman spectra of Bi(Co_0.40_Ti_0.40_Fe_0.20_)O_3_ lead free material.

Fig. 2(a and b) displays the X-ray diffraction (XRD) pattern and Raman spectra of Bi(Co_.40_Ti_0.40_Fe_0.20_)O_3_ at 300 K. Most of the peaks of the pattern are well defined and indexed by using POWDMULT indexing software.^24^ The value of observed and calculated inter-planar spacing of each reflection is in good agreement, showing very small standard deviations (0.0022 Å). As marked in the figure, the all the lattice planes, including major (115), (205), (130), and minor ones satisfy an orthorhombic symmetry. The diffraction pattern of BCTF 80/20 ceramics shows very few impurity peaks with small intensities close to that of the background. The intensities of the corresponding minor peaks are much smaller compared to those of the major peaks of the ferrite structure. The least-squares lattice parameters of the obtained crystal system are: *a* = 9.8750 (22) Å, *b* = 8.4853 (22) Å, and *c* = 18.6033 (22) Å (figure in parenthesis is an estimated standard deviation) and *V* = 1585.82 Å^3^. The crystallite size of BCTF80/20 was calculated using a few high-intensity (major) peaks of the XRD pattern by means of the Scherer formula,^25^*D*_XRD_ = 0.89 *λ*/*β* cos(*θ*) where *β* is the full width at half maxima of intensity, *λ* is the wavelength of X-ray radiation (1.5406 Å), and *θ* = Bragg's angle of diffraction. The average crystallite size was found to be around 30 nm. Comparison of *d*-values of major reflection planes and crystallite size at room temperature are listed in Table 1. Inset of Fig. 2(a) and (b) correlates the room temperature Raman spectra, as well as Gaussian fitted spectra of Bi(Co_0.40_Ti_0.40_Fe_0.20_)O_3_. Raman spectroscopy is a powerful technique for characterization of the molecular (vibrational) structural characteristics of materials. It was utilized to analyze subtle variations in the system of their local structures under an investigation that could be correlated through the structural performance of X-ray diffraction. Theoretical exploration showed eighteen sets of optical phonon modes (4A1 + 5A2 + 9E) for one of the components of solid solution (BFO) that belongs to *R*3*c* space symmetry. Herein, the A1 and E modes are mainly assigned as Raman and IR-active modes while A2 modes belong to the Raman as well as IR inactive modes. To establish the correlation between exact peak frequencies of Raman spectra (different active modes), we applied a Gaussian-fitting procedure for the Raman data. The observed Raman-active modes produce motion of the oxygen ions as well as A/B-site ions in the structure. The observed sharp peaks are at 272, 327, 532, 685, 730, and 863 cm^−1^. All appropriate peaks of BFO have been detected besides an A1 mode, lower than 100 cm^−1^ that could not be identified because of the rejection of the notch filter within the spectrometer.^26^ The Raman modes of BCTF80/20 attribute throughout 200 cm^−1^ due to the bending and stretching modes of an octahedral (FeO_6_) family of perovskite compounds.^27^

**Table 1:** Comparison of *d*-values of major lattice planes with observed relative intensity and variation of crystallite size at 300 K, compound detail – Bi(Co_0.40_Ti_0.40_Fe_0.20_)O_3_, phase: – orthorhombic symmetry

Peak	*d*-Spacing (obs.) (Å)	*d*-Spacing (cal.) (Å)	Relative intensity (*I*/*I*_o_)	*h*	*k*	*l*	Crystallite size (nm)
1	4.2727	4.2427	26	0	2	0	38.08
2	4.1601	4.1595	13	2	1	1	49.41
3	3.8635	3.8626	19	2	0	3	31.88
4	3.6041	3.5952	24	2	1	3	35.15
5	3.2222	3.2211	100	1	1	5	32.08
6	2.9741	2.9715	79	2	0	5	20.46
7	2.7200	2.7191	75	1	3	0	21.42
8	2.5334	2.5361	15	0	1	7	27.40
9	2.3975	2.3983	12	3	2	3	31.88
10	2.2783	2.2820	24	3	2	4	26.36
11	2.1618	2.1706	14	3	3	0	25.86
12	2.1082	2.1077	16	0	4	1	35.15
13	2.0741	2.0748	13	1	4	0	44.01
14	2.0247	2.0243	15	1	4	2	38.08
15	1.9926	1.9926	15	3	2	6	26.36
16	1.9317	1.9319	16	5	0	2	25.02
17	1.8531	1.8534	09	3	1	8	22.08
18	1.7647	1.7642	14	3	3	6	19.03
19	1.7409	1.7409	32	2	0	10	27.43
20	1.6908	1.6912	18	0	0	11	31.08
21	1.6720	1.6725	12	1	5	0	34.26
22	1.6450	1.6455	23	1	3	9	18.98
23	1.5908	1.5908	22	6	0	3	20.16
24	1.4951	1.4938	20	6	2	3	26.88
25	1.4822	1.4822	10	5	1	8	24.06
26	1.4347	1.4348	09	3	5	4	17.80

### 3.2 Elemental and morphological studies

**Fig. 3:**
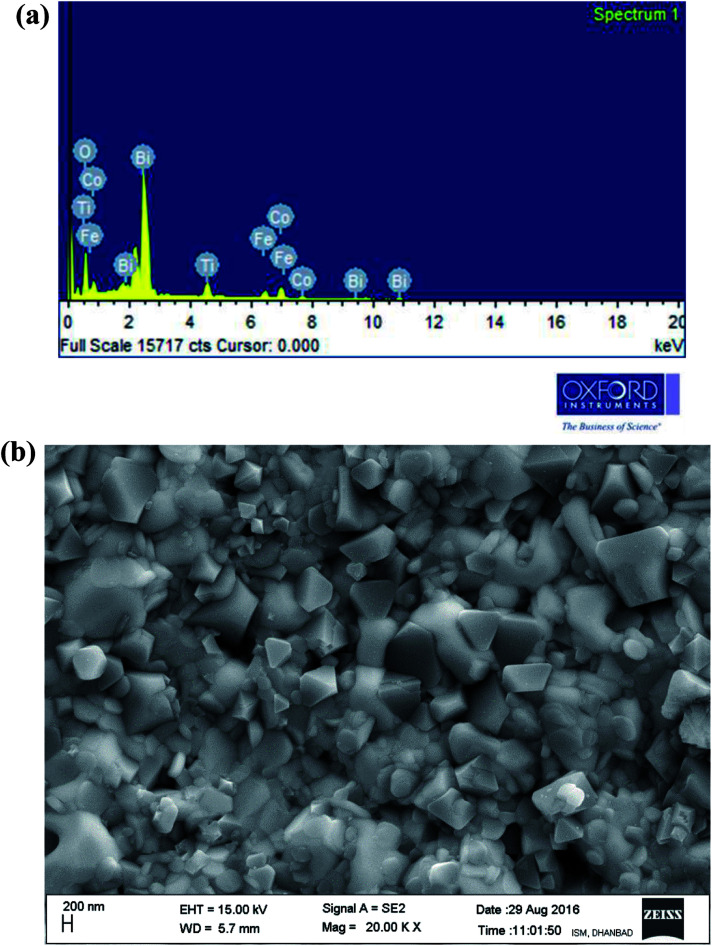
(a and b) EDXMA analysis and corresponding FE-SEM micrograph of as-sintered Bi(Co_0.40_Ti_0.44_Fe_0.20_)O_3_ compound, respectively, at 300 K.

Fig. 3(a and b) illustrates energy dispersive X-ray micro-analysis (EDXMA) and corresponding field-emission scanning electron microscope (FE-SEM) images of Bi(Co_0.40_Ti_0.40_Fe_0.20_)O_3_ at room temperature. The EDXMA analysis provides the presence of bismuth, cobalt, titanium, iron, and oxygen elements with their corresponding atomic ratios close to its stoichiometric concentration. The FE-SEM micrograph shows that grains of BCTF80/20 are of different sizes and shapes, which are distributed homogeneously with few agglomerations. Grains of the said constituents are visibly distinguishable. It is clear that most of the particles are of triangular, spherical, and rectangular shape in morphology with grain sizes between 100–300 nm. However, in bulk BFO, some grains have been fused together to produce a larger size (>50 μm).^28^ By utilizing the profession Image J software,^29^ the grain sizes from micrographs have been calculated. The bulk BFO has relatively large grains whereas the BCTF80/20 sample has smaller grains, but with a highly packed microstructure.

### 3.3 Dielectric studies

A dielectric study is one of the most significant characterizations of as-synthesized specimen material. It provides information about the dielectric permittivity and tangent loss or dielectric loss of the material along with a test of electric field (specific orientation) at operating frequency. The dielectric permittivity is a constitutive component of dielectrics. It has a complex component, which usually would be subject to both operating temperature and frequency variation. Dielectric parameters were examined and recorded with a high precession multimeter attached together to the furnace (temperature-control) and a computer set up. The dielectric constant of the as-synthesized sample was estimated through the general capacitance evaluation at several operated temperatures and frequencies.^30–32^1
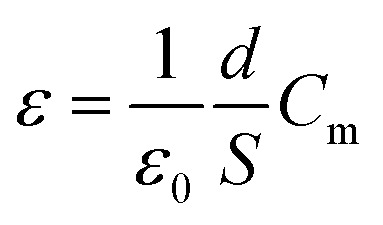
Herein, the symbols display their usual meanings such as; *ε* = dielectric constant, *d* = interfacial thickness of pellet, *ε*_0_ = permittivity of the free space, *S* = specimen pellet sample surface area, and *C*_m_ = measured capacitance in parallel relation.

#### 3.3.1 Temperature dependent dielectric studies

**Fig. 4:**
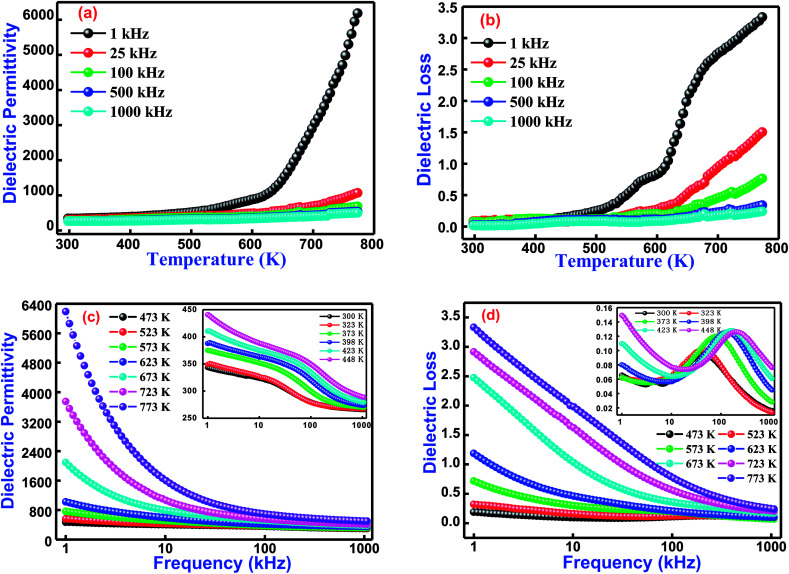
(a) Temperature dependence dielectric permittivity and (b) dielectric losses at several selected frequencies of Bi(Co_0.40_Ti_0.44_Fe_0.20_)O_3_ lead free material. (c) Frequencies dependence dielectric permittivity and (d) dielectric loss nature at various selected temperatures (inset shows lower temperature behaviour) for Bi(Co_0.40_Ti_0.44_Fe_0.20_)O_3_ lead-free material.

Variation of the dielectric constant of cobalt and titanium containing bismuth ferrite [Bi(Co_0.40_Ti_0.40_Fe_0.20_)O_3_] as a function of temperature and frequencies is shown in Fig. 4(a and b). The dielectric properties of said compound depend on its microstructure and synthesis procedure. These properties arise because of an electric dipole moment which establishes the charge transfer between divalent and trivalent cations inside a structure. It is noticed that the appearance of dielectric permittivity undergoes a sharp increase in operating temperature only in the low-frequency zone (<10 kHz), but in the high-frequency zone (25 kHz ≤ dielectric permittivity ≤1000 kHz) an increasing trend is for it to be very slow for all temperatures and to remain almost constant. The low values of dielectric permittivity in this temperature region arise due to grain effects. Upon increasing the temperature, the grain boundaries as well as electrode effects start to dominate their permittivity values. Schottky barriers have created interfaces among grains, surface effects, grain boundaries, and the existence of electrodes in this material. When BCTF80/20 is sintered at an optimized temperature, the outer surface of the pellet sample might receive some oxygen stoichiometry, and resistance becomes higher than with the inner portion of the sample. These Schottky barriers have a much higher capacitance as contrasted towards grains, hence higher values of dielectric constants are detected to pay for the construction of barriers. The higher values of dielectric constants at higher temperatures and low-levels of frequencies generally arise because of the Maxwell–Wagner polarization phenomenon.^33–35^ These higher permittivity values could be ascribed to the presence of a ferroelectric nature in the material.^36^

Additionally, Fig. 4(b) displays the temperature dependent dielectric loss at a few sets of frequency for BCTF80/20. The present trend of spectrum plots is similar to those obtained with dielectric constants. The dielectric loss (spectrum plot trend) arises due to the conduction of smooth curves and relaxation loss (dipole losses) denoted by a peak at assuring temperature. The dielectric loss (spectrum plot trend) introduces several types of loss phenomena. Accordingly, loss due to the dipole reaches its maximum peak point at a certain temperature, and then decreases in the high temperature zone. Actually, with the limitation of the experimental setup, the decreasing trend of the dielectric loss is not measured at additional temperatures. Thus, it is noticed that the dielectric loss has small values at lower temperatures, but further increases are observed with continuous increments of temperature. In addition, the measured values (from Fig. 4(a)) of dielectric permittivity at 1 kHz and 1000 kHz at 300 K and 673 K are 343, 266, 6188, and 501, respectively. Also, the measured dielectric loss values (from Fig. 4(b)) at 1 kHz and 1000 kHz at 300 K and 673 K are 0.065, 0.016, 3.34, and 0.24 respectively.

#### 3.3.2 Frequency dependent dielectric studies

The dielectric behavior as a function of frequency through operating temperature could offer precious information about the nature of the polarization mechanism and localized electric carriers in bismuth ferrite based modified materials.^37^Fig. 4(c and d) correlates the dielectric permittivity and tangent loss *versus* a frequency plot at several temperatures from 300–773 K of Bi(Co_0.40_Ti_0.40_Fe_0.20_)O_3_. The value of dielectric permittivity decreases with a rise of frequency. In the low-frequency region, its value sharply increases, whereas in the high-frequency region it becomes frequency independent at higher temperatures. As the value of dielectric permittivity decreases upon increasing frequency, its nature could be described by Koop's hypothesis.^38^ This also assumes that the nature of variation of the dielectric constant follows a Maxwell–Wagner mechanism.^39,40^ This mechanism has been suggested for an inhomogeneous medium along with the consistence of leading grains isolated through extremely high resistive grain boundaries.^38^ The electric field leads the construction of space charge polarization at the grain boundary. Initially, the grain boundary influence is more dominant throughout the grains, and hence dielectric dispersion occurs in the low-frequency region. Finally, in the high-frequency region, the space charge polarization impact reduces, whereas slow travelling species are not capable to trace an applied electric field. Therefore, as a result, the value of dielectric permittivity decreases.^41^

Fig. 4(d) exhibits the frequency dependence of tangent loss of the Bi(Co_0.40_Ti_0.40_Fe_0.20_)O_3_ compound. The tangent loss is closely associated with the dielectric relaxation process, which represents an energy loss phenomenon of the material. Mostly, the tangent loss phenomenon arises when the polarization lags behind an applied electric field, and hence it is affected by imperfections, grain boundaries, and impurities in the material.^42^ It is also observed, from Fig. 4(d), that tangent loss trends to decrease with a rise in the frequency, and a further increase of frequency gives a small peak (intermediate temperature ranges between 300–448 K) and then decreases again. In conclusion, at higher frequencies, the tangent loss trends to remain constant. Initially, at lower frequencies, highly resistive grain boundaries are much more energetic. As a result, an electronic structure of grain boundaries hinders the orientation of interfacial dipoles. Consequently, with additional electrical energy, absorption occurs during propagation of electrical current through the compound.^43^ Finally, at higher frequencies, the grains become more conductive because of the hopping of charge carriers which are surrounded by B-sites ions; hence, it shows a low tangent loss in the high-frequency region.^44^ Additionally, the compound shows an analogous behavior in permittivity with changes in frequency and temperature. The effects of frequency and temperature on the dielectric parameters are shown in Table 2.

**Table 2:** Comparison of dielectric permittivity and dielectric loss (frequency–temperature dependence) behaviour at an intermediate temperature between 300 K to 773 K for Bi(Co_0.40_Ti_0.40_Fe_0.20_)O_3_ at a particular frequency of 1 kHz and 1000 kHz

Compound	Temperature range	Dielectric parameters at 1 kHz		Dielectric parameters at 1000 kHz	
		Dielectric constant	Dielectric loss	Dielectric constant	Dielectric loss
Bi(Co_0.40_Ti_0.40_Fe_0.20_)O_3_	300 K	343.51	0.07	266.06	0.02
	373 K	375.02	0.06	270.18	0.03
	423 K	410.58	0.11	279.57	0.06
	473 K	471.02	0.19	300.38	0.08
	573 K	764.08	0.72	321.77	0.07
	673 K	2092.87	2.47	387.29	0.17
	773 K	6185.83	3.33	501.02	0.24

### 3.4 Spectroscopic impedance analysis

**Fig. 5:**
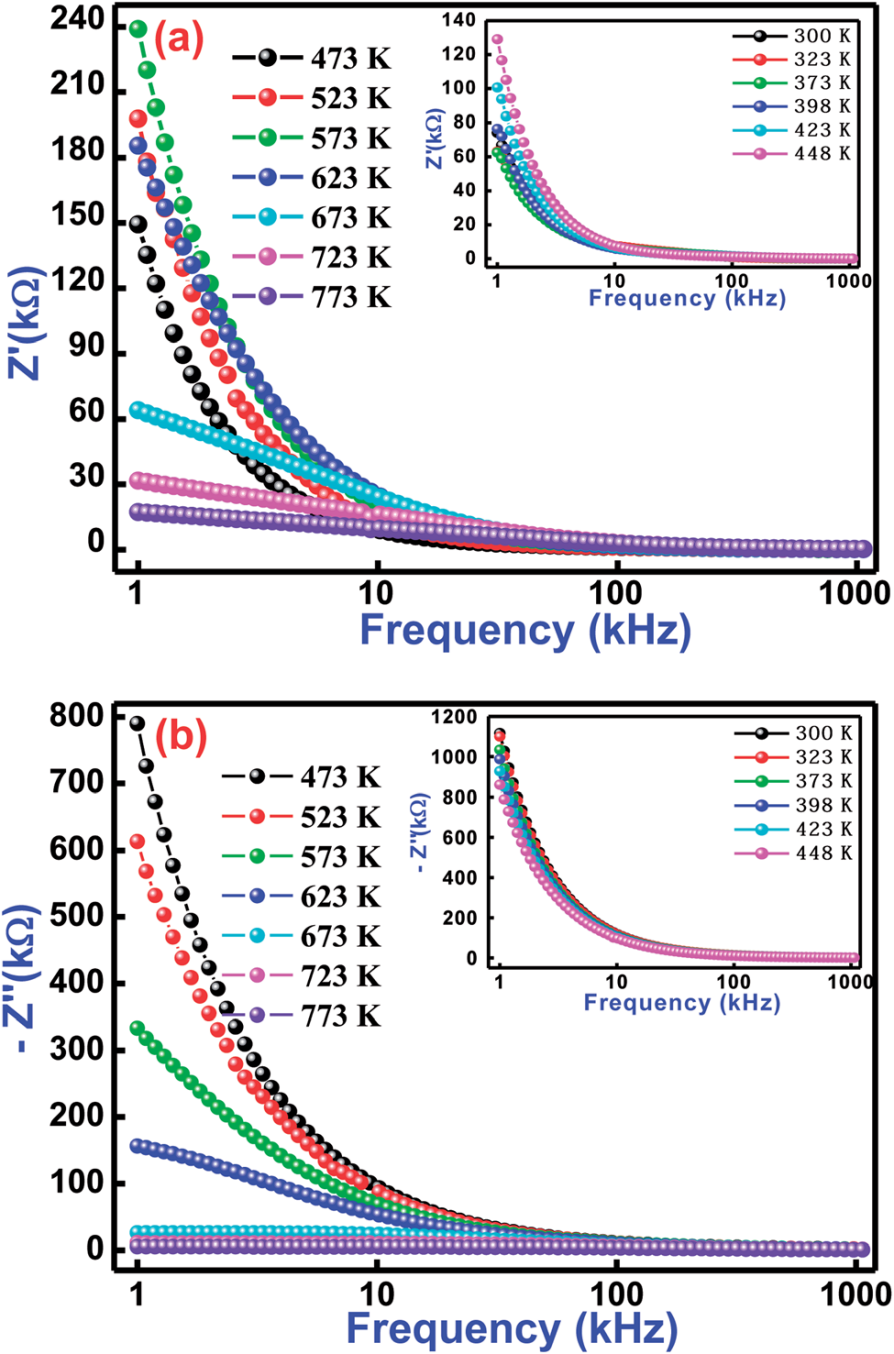
Variation of (a) real impedance (*Z*′) with frequency and (b) imaginary impedance (*Z*′′) with frequency for Bi(Co_0.40_Ti_0.40_Fe_0.20_)O_3_ material at some selected temperatures.

In order to understand physical and electrical characteristics of BCTF80/20, an electrical impedance technique was essentially used.^45–47^ This technique has been found to be useful to establish the correlation between the electrical/conduction mechanism and micro-structure of the study material. Fig. 5(a and b) shows the variation of real impedance (*Z*′) and imaginary impedance (*Z*′′) with frequency at selected temperatures (inset shows low-temperature influence on frequency) for BCTF80/20. It is observed that the magnitude of *Z*′ decreases with a rise in frequency. In the temperature region of 300–623 K, the value of *Z*′ increases slowly but above 623 K, it decreases at low frequencies. Such a decreasing trend of a real component of impedance with rising temperature in the low-frequency region reveals the negative temperature coefficient of resistance phenomenon. For all operating temperatures, the *Z*′ *vs.* frequency plots coincide at higher frequencies, which is mainly due to the release of space charges,^48,49^ and consequently, it lowers the energy barrier at higher frequencies.^50–52^

Fig. 5(b) displays frequency dependence of an imaginary component of impedance (*Z*′′) at selected temperatures. This nature of the spectrum suggests several points, such as (i) the value of *Z*′′ decreases with a rise in both temperature and frequency, (ii) existence of the frequency dependent peaks, (iii) the peak profile is asymmetric, (iv) increase of temperature dependent peak broadening, and (v) coinciding of *Z*′′ peaks at higher frequencies. With increasing temperature, variation of peaks broadening suggests the existence of an electrical relaxation (temperature dependence) process at a particular frequency.^53^ The steady species at the lower temperatures and the defects at higher temperatures are mainly responsible for the relaxation process in the material. It is possible that the relaxation defects or species are liable for the electrical conduction. This conduction may occur due to the hopping of oxygen, ion vacancies, electrons, and defect anomalies surrounded by the localized sites.^54^

**Fig. 6:**
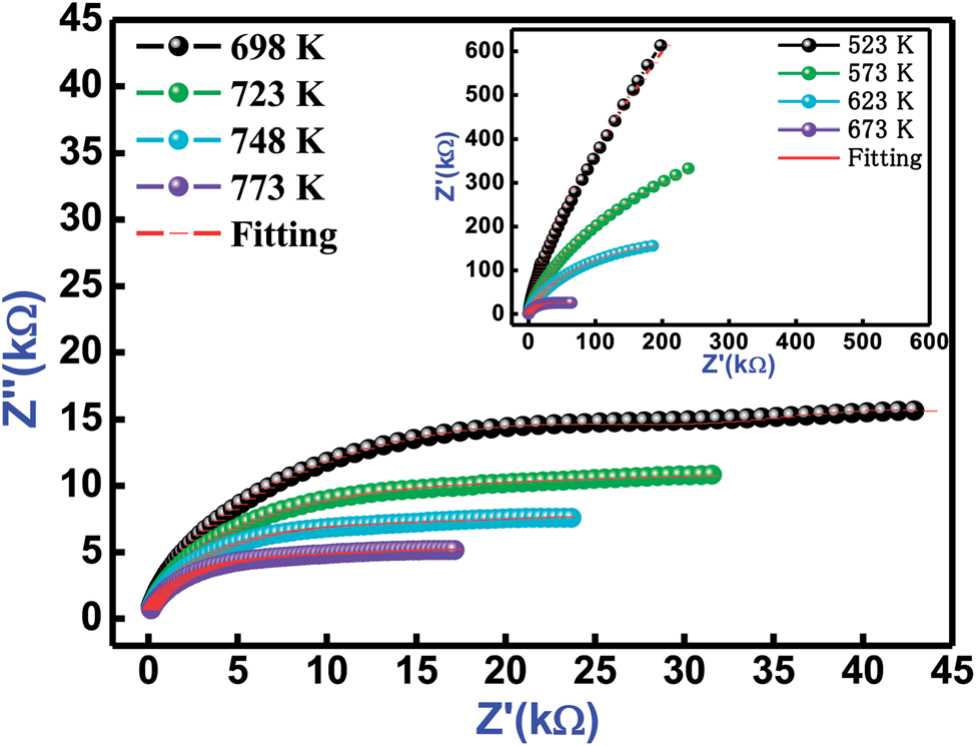
Nyquist plot of Bi(Co_0.40_Ti_0.40_Fe_0.20_)O_3_ material at different temperatures. The red solid line denotes fitting with [(CR) (CQR)] equivalent circuit model.

Fig. 6 refers the Nyquist behaviour (*Z*′ *versus Z*′′ diagram) of Bi(Co_0.40_Ti_0.40_Fe_0.20_)O_3_ in several sets of frequency (1–1000 kHz) and temperatures (523–773 K). In the complex impedance formalism [Z* (*ω*, *T*) = *Z*′ + *iZ*′′], the influence of grain, grain boundary effects, and their electrode contribution need to be studied. The influence of these is clearly seen in a semi-circular arc of the Nyquist diagram, and can be symbolized through an equivalent circuit model. The equivalent model circuit is associated with the parallel combination of a [(CR)(CQR)] electric circuit model that has been used to investigate resistive and capacitive features as a function of temperature. The symbols (circular solid and solid red lines) in Fig. 6 show their measured and fitted values, respectively. By using the fitted plots, the calculated values of bulk capacitance (*C*_b_), bulk resistance (*R*_b_), grain boundary capacitance (*C*_gb_), grain boundary resistance (*R*_gb_), the constant phase element ‘CPE’ and frequency exponent term ‘*n*’ at several temperatures are summarized in Table 3.

**Table 3:** Fitting parameters (*C*_b_, *R*_b_, *C*_gb_ CPE, *R*_gb_, and ‘*n*’) of Bi(Co_0.40_Ti_0.40_Fe_0.20_)O_3_ at several temperatures, model Circuit: [(CR)(CQR)]

Tempt. (K)	*C*_b_ (F cm^2^)	*R*_b_ (Ω cm^2^)	*C*_gb_ (F cm^2^)	CPE	*R*_gb_ (Ω cm^2^)	*n*
523	8.971 × 10^−10^	573.1	1.398 × 10^−10^	3.104 × 10^−9^	1.058 × 10^7^	0.6211
573	7.261 × 10^−10^	1.977 × 10^5^	1.374 × 10^−10^	1.837 × 10^−8^	2.248 × 10^5^	0.5873
623	4.531 × 10^−9^	1.257 × 10^4^	1.303 × 10^−10^	9.637 × 10^−9^	5.176 × 10^5^	0.6201
673	6.793 × 10^−9^	3.373 × 10^4^	1.074 × 10^−10^	6.416 × 10^−9^	6.047 × 10^4^	0.7050
698	9.826 × 10^−9^	1.971 × 10^4^	1.390 × 10^−10^	3.135 × 10^−8^	4.118 × 10^4^	0.5721
723	9.303 × 10^−10^	144.4	1.799 × 10^−10^	1.396 × 10^−6^	8.704 × 10^4^	0.2991
748	1.119 × 10^−9^	86.22	2.009 × 10^−10^	2.214 × 10^−6^	6.783 × 10^4^	0.2831
773	7.726 × 10^−10^	54.21	1.993 × 10^−10^	2.797 × 10^−6^	4.487 × 10^4^	0.2884

### 3.5 Electric modulus analysis

In order to explore the role of electrode polarization, ionic conductivity, electrical and space charge relaxation processes of the sample, a study of one of the impedance-related parameters, complex modulus, is very much required.^55^ This scientific analytical process mainly offers an insight of the electrical relaxation processes arising in the material at various operating frequencies and temperatures. Generally, an electric modulus correlates the relaxation processes when electric displacement remains constant in the material. Therefore, the formalism of the complex electric modulus (M*) is the reciprocal quantity of the permittivity, and physically denotes the real dielectric relaxation phenomenon.

**Fig. 7:**
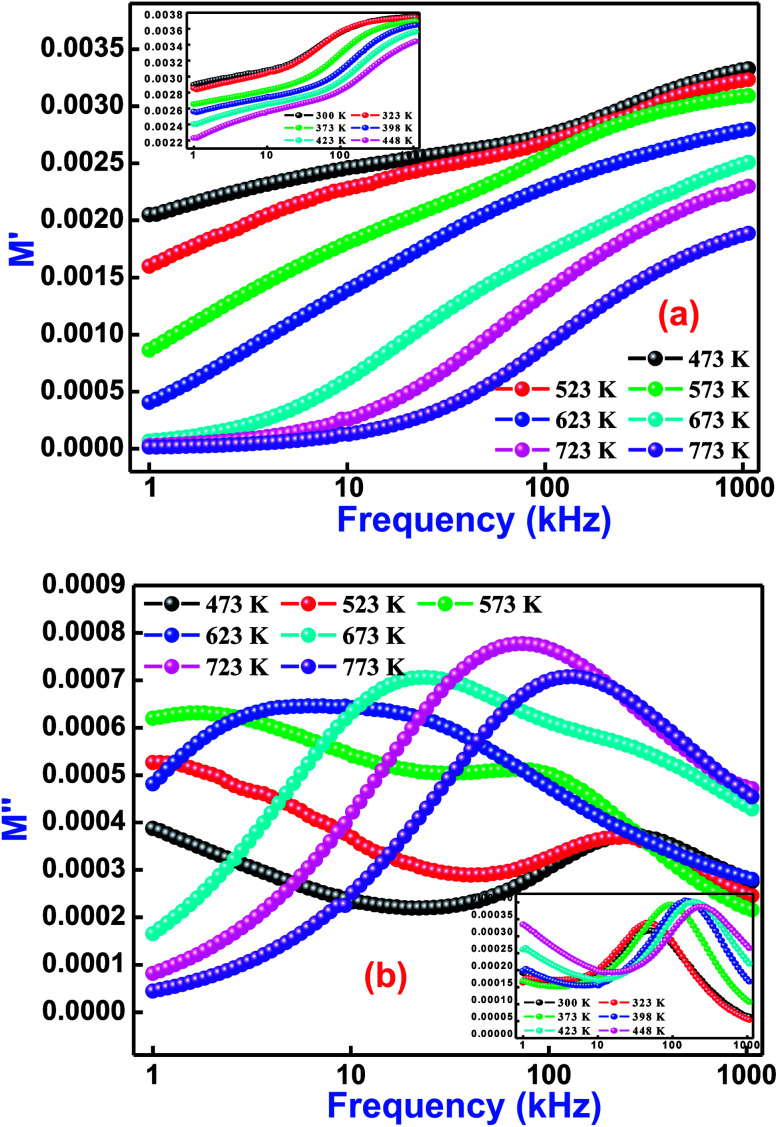
Frequency dependence of (a) *M*′ and (b) *M*′′ for Bi(Co_0.40_Ti_0.40_Fe_0.20_)O_3_ material at various temperatures.

Fig. 7(a and b) exhibits the variation of *M*′ and *M*′′ with frequency at selected temperatures (low temperature variations in the inset of figure) for Bi(Co_0.40_Ti_0.40_Fe_0.20_)O_3_. As observed from this figure (frequency dependent *M*′/*M*′′ modulus spectrum plot), the value of *M*′ increases with increasing frequency, and reaches its maximum value on a further rise of higher frequency because of the presence of relaxation processes. However, the lowest value (nearly zero) of *M*′ appears at lower frequencies indicating the small electrode polarization contribution to *M*′, and therefore it can be dismissed.^56^ The *M*′′ peaks correspond to a certain frequency, (referred as relaxation frequency) in which the charge carriers experience relaxation. In general, for the conduction process relaxation peaks will be detected in the frequency spectra of the *M*′′ part. We observed that the dispersion on *M*′ and *M*′′ plots (through frequency) specify the existence of relaxation time dispersion for conduction. It is clearly seen that the trend of each relaxation peak shifts to the high frequency side with increasing temperature. The peaks specify the transition phenomenon from short range to the long-part mobility through the minimizing of frequencies. However, at lower frequencies, the peak frequency of which the ions are capable move long ranges, while at higher frequencies, ions are mostly held in the potential wells, and are allowed only for localized movement.^57^

### 3.6 Study of electrical conductivity

**Fig. 8:**
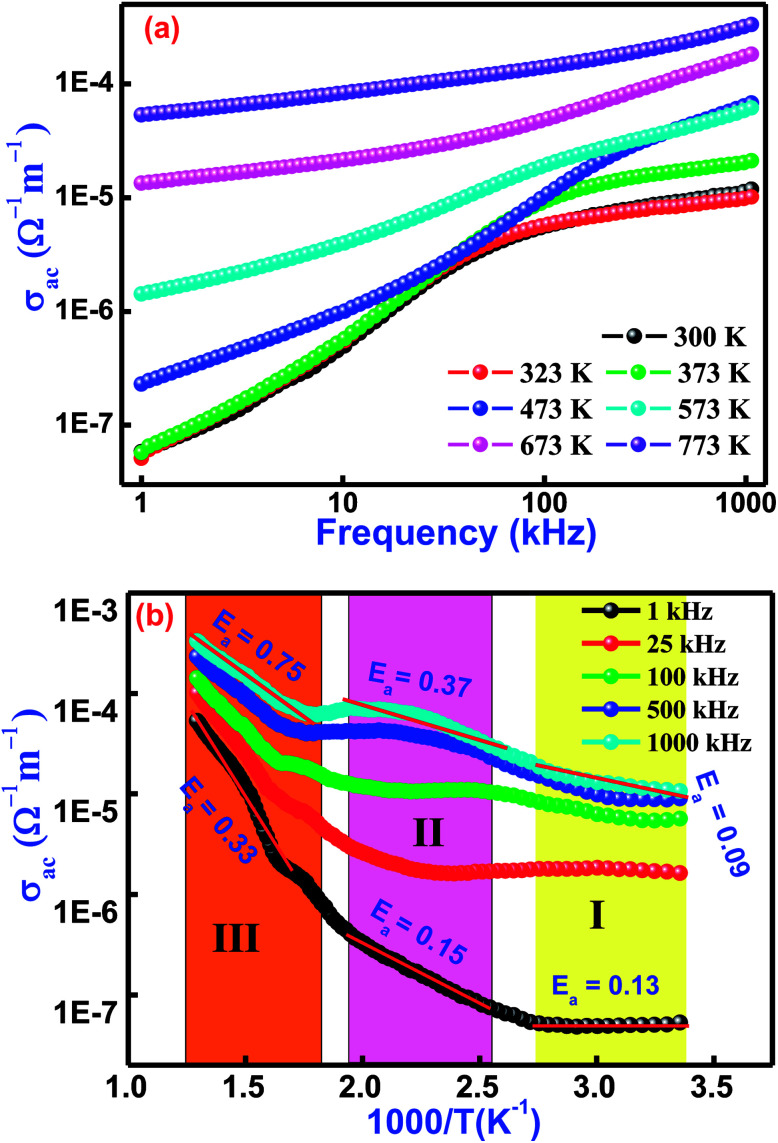
Variation of (a) *σ*_ac_ with frequency and (b) *σ*_ac_ with temperature for Bi(Co_0.40_Ti_0.40_Fe_0.20_)O_3_ lead-free material.

Fig. 8(a) illustrates the frequency variation of ac-conductivity plots of Bi(Co_0.40_Ti_0.40_Fe_0.20_)O_3_ at several temperatures. Generally, the total conductivity of the frequency spectrum consists of the highly dispersive frequency dependent ac-conductivity and frequency independent dc-conductivity. It is observed from the graphs in Fig. 8(a) that in the low-frequency region, the conductivity must be lower at all operating temperatures, while the value of total conductivity systematically increases with an increase of temperature as well as frequency. This trend mostly arises in the low-frequency range because of the existence of the lowest band gap, which is likely to be one factor for a higher value of tangent loss of BCTF80/20. In the high-frequency region, the trend of ac-conductivity plots seems to be a change in slope, which slowly increases with increasing frequency. A particular frequency at which dispersion spectra occurs is known as the hopping frequency that shifts to the high frequency side with rise in temperature.^58^ The AC-conductivity of BCTF80/20 has been calculated in accordance with the impedance data.^59^ In accordance, a suitable formalism specifies the occurrence of a hopping mechanism to the electrical conduction in the compound that has been examined with the following Jonscher's power equation:^60–62^

2 *σ*_ac_(*ω*) = *σ*_α_ + *Aω*^*η*^

where the symbol *σ*_α_ represents a frequency independent part, which provides the value of dc-conductivity, *η* is the frequency exponent term having value between zero and one, and the symbol *A* is a constant (temperature-dependent) that has polarizability strength.

Fig. 8(b) displays the temperature dependent ac-conductivity plots at some frequencies for Bi(Co_0.40_Ti_0.40_Fe_0.20_)O_3_. It was observed that the conductivity increases with rising temperature at all the operating frequencies, which may happen due to the semiconductor nature of the sample in the temperature range of 300–773 K. For a detailed description of the temperature dependent ac-conductivity and activation energy, we subdivided the pattern in three temperature regions, namely Region-I (referred as lower temperature), Region–II (referred as intermediate temperature), and Region–III (referred as higher temperatures). Minor changes in ac-conductivity values are observed in the low-temperature region (Region-I) which increases in the high-temperature region (Region-III) at selected frequencies. Over different regions of temperature, a linear trend of the plot with varying slope is observed. The linear region of the spectrum has been recognized, and the value of the activation energy (*E*_a_) was calculated by utilizing linear the Arrhenius relation, *σ*_ac_ = *σ*_o_ exp(−*E*_a_/*KT*), where *σ*_o_ is a pre-exponential factor as well as a characteristic of the material, *T* and *K* are the absolute temperature and Boltzmann constant, respectively, in different temperature regions (I, II, and III). The variation of activation energy in the different temperature regions can explain the conductivity phenomenon. For better understanding, it is possible to link said regions of conductivity to the movements of charge carriers in Co and Ti containing BFO. A suitable conduction mechanism has already been explained. Therefore, the activation energies of the different regions (region-I, region-II, and region-III) were calculated from the slope of the linear part of plot at 1 kHz which are 0.13, 0.15, 0.33, and 1000 kHz which are 0.09, 0.37, 0.75, respectively. The value of activation energy increases with increasing frequency for intermediate and higher temperature regions (*i.e.*, both Region-II and III). For low temperature (Region-I), the magnitude of activation energy decreases with an increase of frequency. Furthermore, the low value of the activation energy (Region-I) could be ascribed due to the effect of electronic influence on conductivity. Then, the transportation of charge carriers might occur with the hopping of localized states in a disordered system.^63^

### 3.7 Studies of leakage current

**Fig. 9:**
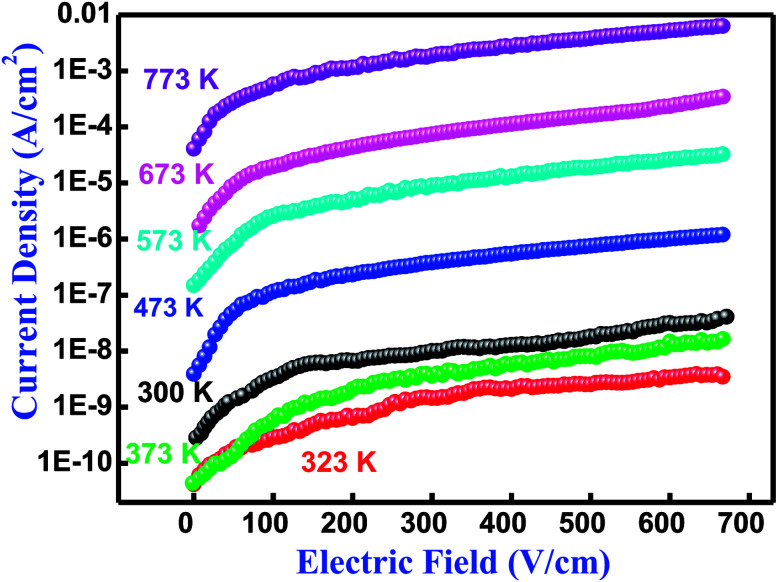
Variation of current density (*J*) with electric field (*E*) relationship at several temperatures of Bi(Co_0.40_Ti_0.40_Fe_0.20_)O_3_ material.

The performance of an electronic material mainly depends upon its insulation characteristics. A good insulation characteristic of materials influences a better performance of the system. There are several experimental procedures for testing insulation and dielectric performance. The leakage current (LC) measurement has been discussed by Fernando and Gubanski.^64^Fig. 9 shows the leakage current behavior of Bi(Cd_0.40_Ti_0.40_Fe_0.20_) at different temperatures between 300–773 K. The current density-electric field spectrum (*J*–*E* spectrum) indicates the low leakage current value of the low electric field region rapidly increases with increasing electric field. In the literature, numerous mechanisms have already been discussed with their possible limitations and elimination processes of leakage current for BFO-related ferroelectric/multiferroic perovskite materials.^65^ In BFO, the leakage current mechanism is dominated by the Poole–Frenkel defects that are similar for few other ferroelectric perovskites.^65,66^ This mechanism contains field supported hopping of charges between Fe^2+^ and Fe^3+^. It is an established fact that in the BFO pure phase, the oxygen vacancies rather than Fe^2+^ ions are generally liable for the higher conductivity, and probably proceed or move as trapping centres.^67,68^ These deeply trapped energy centers are produced by the oxygen vacancies inside the band gap to make activated mobile electrons that contribute to the high conductivity of a sample.^67^ Thus, the remarkable decreasing trend and reasonably low value of leakage current density makes the BCTF 80/20 composition promising for multifunctional devices.

### 3.8 Multiferroic properties

#### 3.8.1 Magnetic features

**Fig. 10:**
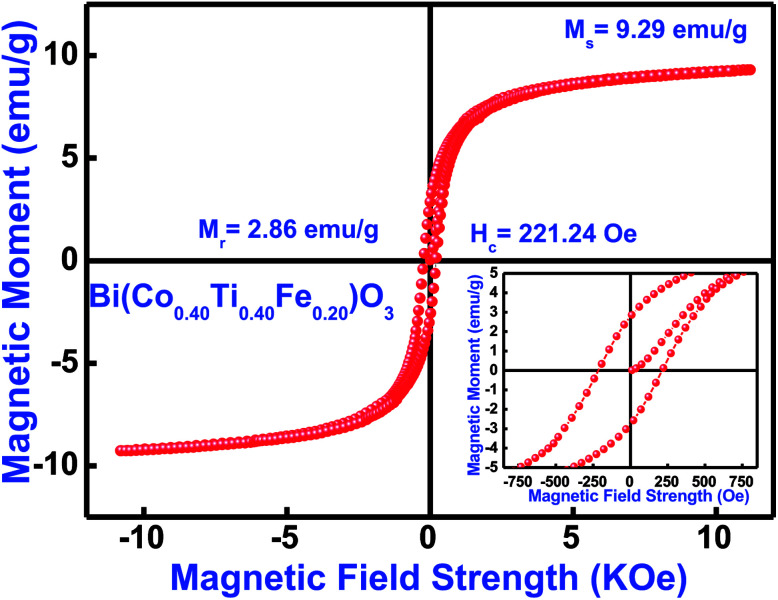
*M*–*H* loops measured at 300 K, (inset) showing enlarged view of Bi(Co_0.40_Ti_0.40_Fe_0.20_)O_3_ material.

Magnetic features of the as-synthesized Bi(Co_0.40_Ti_0.40_Fe_0.20_)O_3_ compound were investigated by using a vibrating sample magnetometer with an application of a magnetic field of ±12 kOe at 300 K. Fig. 10 displays the observed magnetic hysteresis curves of said compound. The magnetic features of multiple additions (Co and Ti) in BFO [*i.e.*, Bi(Co_0.40_Ti_0.40_Fe_0.20_)O_3_] depend on several experimental conditions, such as synthesis technique, cation distribution, particle size, sintering temperature, and so on. The sintered ceramic sample shows the highest value of coercivity (*H*_c_) of 221 Oe, remnant magnetization (*M*_r_) with 2.86 emu g^−1^ and maximum saturation magnetization (*M*_s_) with 9.29 emu g^−1^ [Fig. 10]. Some of the reported results of bulk BFO ceramics depict a typical anti-ferromagnetism without any *M*_r_ with zero.^69,70^ The enhanced value of *M*_r_, *M*_s_, and ferromagnetic ordering could be explained on the basis of an increase in the induced magnetic moment, which strongly affects structural evolution and the difference between ion's radii of B-sites co-substitution. Furthermore, for the formation of oxygen vacancies, a change of valence state from Fe^3+^ to Fe^2+^ is mainly responsible.^71,72^ The occurrence of Fe^2+^ ion may cause the dual exchange interaction between the Fe^2+^ and Fe^3+^ ions along with an oxygen atom; as a consequence, it may enhance the ferromagnetism.^73^ Thus, the minor addition of cobalt/titanium concentration into BFO has significantly enhanced the ferromagnetic parameters.

#### 3.8.2 Ferroelectric *P*–*E* hysteresis loops

**Fig. 11:**
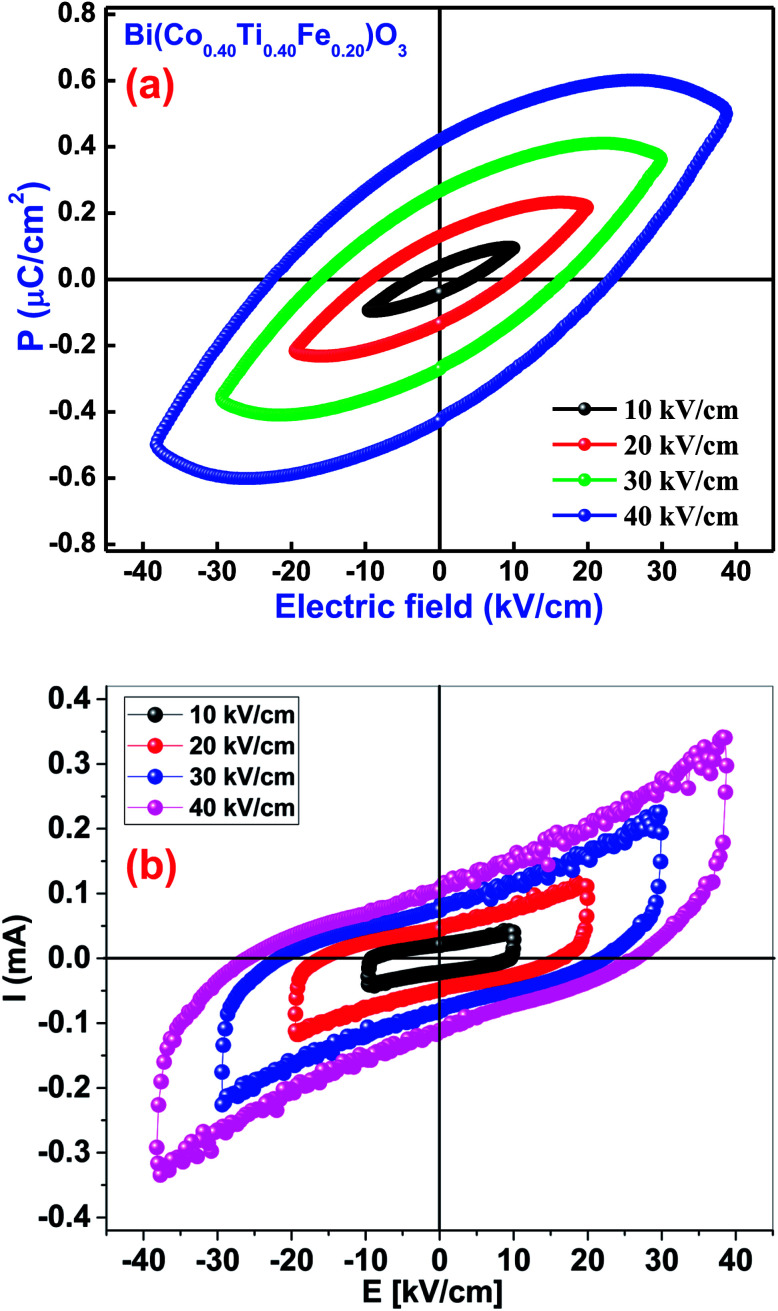
(a) *P*–*E* loops diagram of the Bi(Co_0.40_Ti_0.40_Fe_0.20_)O_3_ material. (b) *I*–*E* loops diagram of the Bi(Co_0.40_Ti_0.40_Fe_0.20_)O_3_ material.

The occurrence of a proper ferroelectric hysteresis loop (polarization *versus* electric field) confirms ferroelectricity of the materials. The impact of this measurement could easily be realized through a polarization (*P*) *versus* electric field (*E*) loop for some capacitor and resistor-based devices. If two components are joined in parallel configuration, we get the *P*–*E* loop shape as an effect of lossy capacitor, wherein, an area within the loop is directly proportional to the tangent loss of the device parameters, and its slope is proportional to capacitance. To examine the ferroelectric features of Bi(Co_0.40_Ti_0.40_Fe_0.20_)O_3_, the *P*–*E* hysteresis loops were traced at a particular frequency of 1 kHz in presence of different applied electric fields [Fig. 11(a)]. The ferroelectric nature of pure BFO was observed because of the presence of 6 s^2^ lone-pairs from the Bi^3+^cations. BCTF80/20 exhibits a high remnant polarization of 0.42 μC cm^−2^. It is to be mentioned that it is difficult to achieve a well-saturated ferroelectric loop since the leakage current influence is much larger. The value of remnant polarization of the said study compound increases with an increase of applied electric field (from 10 kV cm^−1^ to 40 kV cm^−1^). During the measurements, higher electric fields could not apply due to the presence of high leakage-current density of BCTF80/20. The current *vs.* electric field also can be used to study the ferroelectric behavior of ferroelectric ceramics as reported by Kumar *et al.* for PLZT ceramics.^74,75^Fig. 11(b) shows the *I*–*E* loops for the BCTF80/20 ceramics. It is evident from Fig. 11(b) that leakage current is dominating the domain switching current and increases with an increase in an applied electric field. A domain switching peak is not visible due to high conducting nature of the ceramics.

## 4. Conclusion

In summary, a lead-free pure phase multiferroic material of composition Bi(Co_0.40_Ti_0.40_Fe_0.20_)O_3_ was successfully synthesized by a solid state reaction technique. The structural (phase identification and basic structural) studies of the sample confirmed the formation of a pure phasic system by using powder X-ray diffraction. The lattice parameters, average crystallite size, cell volume, and micro-strain value are strongly affected by addition of cobalt and titanium into the bismuth ferrite. The FE-SEM micrograph suggests that the grains were aggregated in triangular, spherical, rectangular shapes together in a nano-meter range. EDXMA analysis confirmed the presence of all elements in the sample near their stoichiometric concentrations. The dielectric parameters (dielectric and loss tangent) were established to be improved due to the elimination of oxygen vacancies with significant doping/substitution. The ac-conductivity analysis was investigated in an extensive range of frequency (1–1000 kHz) and temperature (300–773 K). The temperature dependence of conductivity analysis suggests that the hopping charge carriers are dominant at low temperatures, whereas oxygen vacancies are dominant at intermediate and high temperatures. The significant enhancement in the remnant polarization and saturation magnetization values of BCTF80/20 ceramics may offer a promising candidate for the development of new generation electronic devices.

## Conflicts of interest

The authors declare that they have no conflicts of interest.

## Supplementary Material
